# The curious physician: exploring the role of curiosity in professionalism, patient care, and well-being

**DOI:** 10.1080/07853890.2024.2392887

**Published:** 2024-08-19

**Authors:** Till Johannes Bugaj, Tim Alexander Schwarz, Hans-Christoph Friederich, Christoph Nikendei

**Affiliations:** Department of General Internal Medicine and Psychosomatics, University of Heidelberg Medical Hospital, Germany

**Keywords:** Physicians, exploratory behavior, burnout, burnout prevention, physician health, well-being, medicine, medical education

## Abstract

**Introduction:**

Curiosity is a fundamental human trait that drives learning and exploration. However, research on curiosity has received little attention in the medical field, despite its potential to enhance knowledge acquisition, work performance, and psychosocial well-being. This study aimed to address part of this gap by investigating physicians’ perspectives on their personal experiences with curiosity and its role in their professional practice and medical training.

**Materials and Methods:**

This qualitative study was conducted with 12 physicians from the University of Heidelberg Medical Hospital. Participants were contacted randomly *via* email and invited to participate in the study. Data were collected through semi-structured interviews between September 2019 and February 2020. The authors employed Mayring’s approach, which denotes a qualitative content analysis method characterized by its systematic and rule-guided approach to analyzing textual data, aiming to extract meaningful insights and patterns or themes. The identified themes were linked to overall categories to draw conclusions from the data.

**Results:**

The interviewees highlighted three main areas regarding curiosity’s importance [1]: as a driving force for (lifelong) education [2], in building empathetic physician-patient relationships, and [3] as a core quality of a good researcher. They primarily linked curiosity with positive emotions, while the non-expression of curiosity was associated with dissatisfaction, boredom, and exhaustion. Factors such as heavy workloads, time constraints, stress, and lack of autonomy inhibit their curiosity, while varied activities, professional exchange with colleagues, and exposure to new challenges foster it. Physicians’ perspectives on the link between burnout and curiosity were not consistent. Interestingly, some viewed curiosity as protective against burnout, while others saw excessive curiosity as a potential source of frustration and burnout.

**Conclusion:**

This study represents the first attempt to explore physicians’ perspectives on curiosity in medicine. The findings highlight the potential importance of curiosity in shaping medical professionalism and improving patient care. However, its pursuit is hampered by the challenging working conditions faced by doctors, suggesting a need for enhanced support and cultivation.

## KEY MESSAGES

1. Physicians identify curiosity as a significant factor in increasing their engagement with medical knowledge, improving patient care, and fostering empathetic doctor-patient relationships.
2. External factors such as time constraints and stress emerge as predominant barriers to physician curiosity, highlighting the importance of addressing systemic challenges to support curiosity.
3. Physicians express a nuanced view of the relationship between curiosity, well-being and burnout, suggesting the need for deeper investigation.

## Introduction

Curiosity is the urge to learn new information and seek out new sensory experiences, which stimulates exploratory behavior [1–3]. It is a fundamental component of human nature. We all experience it throughout our day browsing the internet, listening to new music or gossiping with colleagues, while it also drives the human capacity to adapt to new problems [4]. Therefore, curiosity is an essential factor in scientific discovery and a driving force behind the advancement of our species [5].

Research highlights the importance of curiosity in both the workplace and in education. It has been shown to be of great value in academic settings [6–8], e.g. with positive effects on knowledge acquisition [9–11] and lifelong learning [12]. A study with 320 workers demonstrated that curiosity had a predictive value that was not captured by 12 other measures of job performance [13]. Additionally, a questionnaire study found that curiosity mediated the effects of conscientiousness and openness to experiences on learning among physicians [14].

The medical profession is characterized by high-stress environments, long working hours and a high prevalence of burnout [15,16]. Curiosity is positively correlated with well-being, quality of life and proactive-socialization behavior [17–19] as well as with personal growth and life satisfaction [20–22]. It has been postulated as a possible protective factor against stress and burnout [23,24], however, it is also linked to perceived workload [25].

Faith Fitzgerald recognized the importance of curiosity in medical professionalism over 20 years ago: ‘I believe that it is curiosity that converts strangers (the objects of analysis) into people we can empathize with. (…) Both the science and the art of medicine are advanced by curiosity’ [26]. Indeed, curiosity is one of the most prevalent motivations for studying medicine [27] and medical students closely link their anticipated job satisfaction to the degree to which they expect their curiosity to be fulfilled by working as a physician [28]. However, Faith Fitzgerald diagnosed that curiosity is at risk among medical students and physicians, attributing this issue to medical education suppressing rather than nourishing curiosity [26]. Many doctors and medical educators have picked up her call and urged for more curiosity in clinical medicine, medical training, and research [29–33]. Despite this, research on curiosity in physicians is lacking. The authors were only able to identify one empirical study of curiosity in physicians [14], underscoring that curiosity is disregarded in the medical field.

Although opinion pieces argue for greater attention to curiosity in the medical environment, no study has yet investigated physicians’ views on curiosity in their workplace. Such exploratory research is typically qualitative, aiming to capture the (free) associations and thoughts of the subjects as openly as possible. This study aims to fill part of this research gap through the qualitative approach, providing a starting point for the design and planning of future research efforts. The authors therefore explore physician’s viewpoints on (I) curiosity’s role in the development of professionalism and patient care in clinical medicine, (II) their personal experiences with and cultivation of curiosity and impact on well-being, (III) curiosity’s significance in medical education from the learner’s and teacher’s perspective.

## Materials and methods

This interview study was conducted between September 2019 and February 2020 at the University of Heidelberg Medical Hospital, one of the largest German hospitals. The researchers carried out semi-structured interviews with twelve physicians who had an average age of 33.1 years and an average of 6.5 years of clinical experience. All participants were provided with a detailed explanation of the study and their written consent was obtained before the interviews began. Written consent was also given for the publication of quotes from the interview. Voluntary participation, confidentiality of the data and anonymity were emphasized and no incentives were given. The study was conducted in accordance with the principles of the Declaration of Helsinki (64th WMA General Assembly, Fortaleza, Brazil, October 2013) and approved by the Ethics Committee of the University of Heidelberg (No. S-592/2019).

### Study design and setting

This qualitative, descriptive, and exploratory study utilized a blend of deductive and inductive methods. A semi-structured interview (SSI) guide was formulated based on a literature review and theoretical considerations by the researchers, aligning with the methodological approach outlined by Helfferich [34]. This guide informed the overarching categories for data analysis. The interviews were then analyzed using Mayring’s method of qualitative content analysis [35,36], employing an inductive process to identify emerging themes within the data. This research design was selected because it offers a balanced approach integrating deductive and inductive methods. Predetermined questions ensure that key areas of interest, facets of curiosity in a physician’s work life and medical education, are covered, whilst the subjective perspectives of the interviewees are captured in the themes emerging from the data. Mayring’s method provides a structured and systematic approach to qualitative content analysis and is very established in the German language region. The SSI were carried out between September 2019 and February 2020 at the University of Heidelberg Medical Hospital. As it is typical for a University Hospital, physicians usually conduct research and teach students from the affiliated University, additionally to their clinical work. Nine of the interviews were conducted face-to-face in the hospital, three *via* telephone. The research findings were presented adhering to the guidelines outlined in the Consolidated Criteria for Reporting Qualitative Studies (COREQ) checklist [37].

### Participants

In an initial recruitment phase *n* = 20 physicians of the University of Heidelberg Medical Hospital were randomly contacted by e-mail and invited to voluntarily participate in the study. The sole exclusion criterion was a lack of fluency in the German language; however, this was not an issue for any of the physicians contacted. The age of the participants ranged from 26 to 41 years, with a mean age of 33.1 years and they had an average of 6.5 years of clinical experience. Five participants were female and seven were male (for details see Table 1). Participation was voluntary.

**Table 1.** Sociodemographic data.

Gender	5 female (42%), 7 male (58%)
Age	33.1 (26 – 41 years old)
Average work experience in years	6.5
Specialty	Gastroenterology (*n* = 2) General Internal Medicine (*n* = 2) Cardiology (*n* = 2) Endocrinology (*n* = 1) Nephrology (*n* = 1) Obstetrics (*n* = 1) Psychosomatic Medicine (*n* = 1) Visceral Surgery (*n* = 2)

### Conceptual framework

This study was based on an interpretive framework [38,39]. The methodology prioritized understanding physicians’ subjective experiences with the pursuit of curiosity and its impact on well-being, as well as their perspectives on its role in their professional lives and medical education. By adopting an interpretive lens, the researchers aimed to explore the intricate interplay between the constructs under investigation, recognizing the complexity inherent in human experiences within the medical profession. This framework guided data collection and analysis, with the goal of capturing diverse perspectives of participants and uncovering nuanced insights.

### Semi-structured interview (SSI) guideline

The authors Dr. med. Till Johannes Bugaj (TJB, male), apl. Prof. Dr. Christoph Nikendei, MME (CN, male) and Tim Alexander Schwarz (TAS, male) developed the SSI guideline based on literature research, identifying key themes and concepts relevant to the study and with the methodological approach by Helfferich [34]. The interviews consisted of leading questions, and if required, some clarifying and probing questions could be added. All questions underwent a piloting process where the researchers conducted three interviews with physicians from the University of Heidelberg Medical Hospital randomly contacted by email. Using the think-aloud technique [40], participants were asked to verbalize their thought process while answering the questions. This method aims to gain a better understanding of how interviewees interpret the questions and to identify any ambiguities. The responses were audio recorded and stored securely. Consequently, adjustments were made to the SSI guide. For example, the question ‘Do you think that curious doctors burn out less often?’ was replaced with ‘What kind of connection might exist between curiosity and burnout?’ because respondents preferred a more open-ended question. Table 2 shows the leading questions of the final SSI guideline.

**Table 2.** Semi-structured interview guideline.

Category	Question
(I) Curiosity’s role in the development of professionalism and patient care in clinical medicine	Do you consider curiosity to be a central quality of a good doctor?
	Do you think that patients benefit from curious practitioners?
	Can you think of specific clinical cases that were solved through curiosity?
(II) Doctor’s personal experiences with and cultivation of curiosity and impact on well-being	Has your curiosity for medical topics changed from the first day in medical school until today?
	Can you satisfy your curiosity in your everyday professional life?
	How did you feel during periods of training where curiosity was lacking?
	How does your stress level influence your sense of curiosity?
	What kind of connection might exist between curiosity and burnout?
(III) Curiosity’s significance in medical education from the learner’s and teacher’s perspective	If you could make one change that would lead to more room for curiosity in medical training: what would that be?
	What already exists in medical education that fosters curiosity?
	Have you ever noticed a lack of curiosity among the students you supervise?

### Data collection procedure

The physicians who agreed to participate in the study were met individually between September 2019 and February 2020. They were interviewed once by TAS (doctoral student), who had been trained to conduct these interviews and was supervised by two physicians and experienced researchers (TJB and CN), utilizing the SSI guideline. TAS had no prior contact with any of the participants and they only knew that he was a doctoral student (no knowledge of his assumptions or interests in the research topic). The subjects were informed about the background and aim of the study. At the beginning of each interview, participants completed a questionnaire asking for sociodemographic information and previous work experience. New subjects were recruited until no new content appeared in the interviews (data saturation). No additional field notes were required in any of the interviews conducted. The interviews were audio recorded and then transcribed verbatim by the interviewer. The interviews and transcripts were securely stored offline in password-protected, pseudonymized files on two research group computers. Transcripts were not returned to participants for comment. The medium length of the interviews was 14.6 min (8–31 min). The researchers embraced a reflexive approach, recognizing that their personal biases and background played a role in shaping both the collected data and its interpretation [41]. Being white, German men, they acknowledged that their notions about curiosity in the medical profession might differ from other perspectives. They also recognized that the positionality of the interviewer, as a junior researcher with limited clinical experience, interacting with more senior clinicians, as well as the positionality of the research team, as colleagues of the interviewees working at the same hospital, may have affected the ways they shared their experiences and opinions.

### Data analysis

For the analysis of the interviews, a qualitative content analysis according to Mayring [35,36] was conducted using the application MAXQDA 11 (VERBI Software, 2015 - a copyright license was held by the authors). First, the interviews were read by TAS and TJB several times to get an overall understanding of the content. The main categories were deductively derived from the SSI guide and formed the basis of the coding template. Then, Mayring’s ‘inductive category formation’ [36] method was applied, which emphasizes the emergence of themes from the material itself. The smallest unit of analysis was determined to be a single sentence, and the longest a full response to a question. These units were independently labeled by TAS and TJB with codes reflecting their content. Codes with related meanings were grouped under broader themes, which were then sorted to the main categories. After every third interview, TAS and TJB engaged in an iterative process of investigator triangulation to discuss discrepancies in their coding, merging related themes, and adjusting the coding template accordingly. A third researcher (CN) was consulted to resolve any disagreements. The final coding template was subsequently applied to all 12 interviews. Participants did not provide feedback on the findings. Mean values for sociodemographic data, such as age and work experience among the participants, were calculated using SPSS25 (IBM, 2017 - a copyright license was held by the authors) and are presented in Table 1.

## Results

Out of the physicians approached in the first recruitment phase, *n* = 16 (80%) were willing to participate. However, data saturation was already reached after *n* = 12 interviews, when the last two interviews had not revealed any changes to the themes, new themes or new perspectives on the existing categories. As a result, no second recruitment phase was necessary. In the qualitative content analysis 244 single codes were inductively extracted from the transcripts, and nine themes were identified. These themes were then classified according to the three main categories, which were derived from the interview questions: (I) Curiosity’s role in the development of professionalism and patient care in clinical medicine, (II) doctor’s personal experiences with and cultivation of curiosity and impact on well-being, and (III) curiosity’s significance in medical education from the learner’s and teacher’s perspective (see Table 3).

**Table 3.** Exemplary quotes from the participants for each theme.

Category	Theme	Exemplary quote with the participant number in brackets
(I) Curiosity’s role in the development of professionalism and patient care in clinical medicine	Curiosity leads to more engagement with medical knowledge and patients	‘So, without this drive to learn (…) I don’t think you would develop further and would not be able to master certain situations’. (#10)
		‘Anyone who suffers is better off if they can talk about their suffering and someone listens to them. If the listener isn’t curious, this will certainly be noticeable to the (…) patient through body language and what he’s saying and will be less curative. I think everyone who communicates is happy when the other side (…) is curious and inquisitive’. (#7)
		‘I think, in a broader sense, curiosity is of course also an important characteristic of a physician, especially for the scientifically active physician, who naturally wants to find out how diseases arise, why they arise, how and why to treat them, how this works, how it works better, how to optimize medical learning and action. But I don’t think that’s limited to the academically active physician, everyone needs it’. (#9)
	Curiosity is beneficial in (unusual) clinical patient cases	‘(…) you could have stopped earlier and sent the patient home simply with antibiotics, but, yes, if we hadn’t had this curiosity to continue investigating together with the senior physician on the ward, then it probably would have fallen short, yes’. (#10)
		‘If someone shows up for example with a skin rash, that doesn’t itch - I don’t know what it is. (…) then of course it’s important that you are curious (…): What does the efflorescence look like? That you look at the whole body (…), that you take a look into the throat, are there any indications, for example, for a former tonsillitis? (…) thus, you arrive at a working hypothesis and conclude, ‘ok, that could be a streptococcus (…) induced skin rash’ (#5)
(II) Doctor’s personal experiences with and cultivation of curiosity and impact on well-being	*Curiosity evolves through the course of one’s career*	‘I would actually claim, at least for my part, that curiosity really does become more specific, that it develops in niches that simply interest you, and that this general curiosity - which you need in the beginning because otherwise you won’t really make it as a doctor - is decreasing, honestly’. (#6)
		‘I believe that that [curiosity] is changing especially now in the context of working as a doctor, yes. More towards the negative (…) I think, in the long run, you acquire blinders (…)’. (#3)
		‘Clinically, I have discovered a few focal points for myself over the years, where my curiosity has increased disproportionately in these areas in which I already know my way around very well. Because, if you have already worked a lot on it, every news is infinitely exciting - so that I would say that overall curiosity has increased slightly’. (#7)
	Working conditions mostly inhibit personal curiosity	‘(…) It’s not untypical to say. ‘Ok, regarding the economy of time, I don’t have time for that. I’m turfing that to the specialty where I think it belongs, e.g. dermatology’. And that’s actually the direction the system is pushing towards. That means that the curiosity you have isn’t necessarily rewarded by the medical system you’re in, (…) but on the contrary, you lose time if you get too involved with things that are outside the scope of your narrow specialty’. (#5)
	The attitudes of the doctors matter	‘And accordingly, perhaps a person who is curious is also at the same time a person who attaches value to things, and perhaps this is (…) bitterly necessary in your life as a doctor: that you attach value to things’. (#5)
	Fulfillment of curiosity elicits positive emotions, inhibition of curiosity negative emotions	‘This also has to do with the fact that it’s fun when it makes you curious (…) I think it’s simply linked to doing a better job’. (#1)
		‘Curiosity is also exhausting, curiosity means that I want to understand something, that I am interested in something, somehow looking for resources that inform me, contacting people, searching the internet, et cetera. That means, if I am curious and looking things up, my working day will be longer’. (#6)
		‘In everyday professional life, I find it unsatisfactory if you don’t have time to read and study and to better understand things that interest you’. (#7)
		‘Well, degeneration, right. I remember my time in orthopedics, I did my practical year there, right. It was a real catastrophe (…) I think that makes you depressed in the end (…). And, it also leads to the fact that one simply thinks (…) more shallowly, that one (…) becomes jaded’. (#4)
	*The relationship between burnout and curiosity is complex*	‘I do think that (…) curious doctors tend to burn out less (…) that [curiosity] is just like a little reward (…). I believe that curiosity (…) creates its own very intrinsic motivation, (…) and I believe that it protects you’. (#4)
		‘I think burnout is always (…) a problem that arises when expectation and reality do not match. If you’re actually extremely curious, but never manage to pursue things and have no (…) means and no time for it, I can imagine that it promotes burnout (…) if you are stuck in a professional treadmill, that you’d rather like to get out of and do other things’. (#9)
		‘At some point you have no strength left for your curiosity and you aren’t able to pursue it anymore’. (#12)
		‘I’d rather adduce an example (…): the flame. You can either burn an entire forest with it or you can put it in an oven and thereby warm a house and cook with it. Thus, that’s a very essential question: ‘how do I handle this energy?’ So, it can be both’. (#5)
(III) Curiosity’s significance in medical education from the learner’s and teacher’s perspective	*Physicians lament a lack of curiosity in students*	‘In that [curiosity] regard people [students] are really very different. (…) it’s more like an attitude, like a good soldier who can overcome a situation that he doesn’t really like. But I don’t think that’s where the living art of our field begins’. (#5)
	*Curiosity in medical students and physicians can be nurtured with the right methods and in the right conditions*	‘(…) interactive formats such as pbl [problem-based learning], where things are worked out together, where, in a dynamic process, you can observe how curiosity increases. Every patient contact for someone (…) in medical education can increase curiosity. And today’s possibilities for tapping into pubmed’s infinite resources of knowledge’. (#7)
		‘The fact that almost everyone comes in contact with research at some point during their education (…) is definitely something that encourages to dive in deeper’. (#11)
		‘I would create time and space for casuistry. Where it is absolutely clear that what the doctor experiences and sees can be discussed in a medical group, or at least with experienced doctors, so that curiosity is really satisfied’. (#5)

### Category I: curiosity’s role in the development of professionalism and patient care in clinical medicine

#### Theme: curiosity leads to more engagement with medical knowledge and patients

The interviewees attributed numerous positive effects to curiosity, describing it as a ‘driving force’ that leads doctors to acquire broader and deeper knowledge, study and train more rigorously and ultimately provide better patient care. This was viewed as an aspect of lifelong education. In doctor-patient interactions, curiosity was seen as a prerequisite for taking thorough medical histories and adopting an empathetic approach with patients. It was commented multiple times that when healthcare providers exhibit genuine curiosity, patients will feel taken more seriously and more comfortable. Furthermore, curiosity was highlighted as a crucial quality, or even a necessary requirement, for good researchers.

#### Theme: curiosity is beneficial in (unusual) clinical patient cases

Asked to reflect on specific clinical cases in which curiosity played a role, the doctors often cited examples involving rare illnesses or unusual courses of disease. The solution to these cases was attributed to the medical personnel’s broad knowledge and their determination to solve the case, both rooted in curiosity. Several participants drew a parallel to the TV Show ‘House, M.D.’ to illustrate their points. One physician attributed the discovery of a rare cancer in a patient directly to the curiosity that drove extensive testing. Additionally, curiosity was described as playing an important role in everyday diagnoses.

### Category II: doctor’s personal experiences with and cultivation of curiosity and impact on well-being

#### Theme: curiosity evolves through the course of one’s career

The doctors reported changes in their curiosity throughout their medical studies and professional careers. Some described a decrease in their curiosity about ‘medicine as a whole’, in favor of an increase in curiosity about specific areas or topics. Conversely, others reported an overall increase in curiosity due to a focus on specific aspects related to their specializations.

#### Theme: working conditions mostly inhibit personal curiosity

One focus of the interviews was how external factors affect curiosity. While a few curiosity promoting factors were mentioned, many more inhibiting it were identified. The hospital’s working conditions were a common theme, with interviewees highlighting the stressful work environment and lack of time as major obstacles. Many participants expressed that the system they worked in disincentivized the pursuit of curiosity due to the time or resources it would cost. Factors such as a lack of autonomy and excessive routine work were also identified as hindering curiosity. Physicians expressed feeling a pressure to prioritize and focus on topics that were acutely relevant to their clinical work if they were to pursue curiosity at all. When asked what promotes their curiosity, many physicians foremost suggested alleviating the negative influences, like a lack of time and stress. They also mentioned that variety in their work, professional exchange with colleagues, and facing new challenges support curiosity.

#### Theme: the attitudes of the doctors matter

Curiosity appears to be influenced by both external factors and the doctor’s attitude. Unusual factors such as sympathy for a specific patient or valuing the ‘narcissistic reward’ of putting in extra effort can also play a role in how much a physician pursues their curiosity.

#### Theme: fulfillment of curiosity elicits positive emotions, inhibition of curiosity negative emotions

Fulfilling curiosity in everyday work was noted as evoking positive emotions such as enjoyment and gratification. Conversely, suppressing curiosity could lead to dissatisfaction and boredom. Physicians described phases during which they were unable to pursue their curiosity for an extended period as dull, restricting and exhausting. A mutual condition of curiosity and stress was also identified: Some interviewees perceived the suppression of curiosity as stress-inducing, and high stress levels led to a reduction of curiosity.

#### Theme: the relationship between burnout and curiosity is complex

Participants had varying views on the connection between curiosity and burnout. Some saw curiosity as protective, others as a risk factor. One interviewee used a metaphor to express this potentially ambivalent relationship, with curiosity being a ‘flame’ that could either fuel an oven or burn down a forest.

Physicians viewing curiosity as protective against burnout, believed that intrinsic motivation resulting from curiosity and the gratification of satisfying curiosity, could shield against challenging work environments and frustrations. Additionally, higher curiosity was linked to prosocial behavior, ultimately proving beneficial in warding off burnout.

Conversely, several participants feared excessive curiosity encouraging burnout if it resulted in overburdening. Insufficient satisfaction of high curiosity was postulated to lead to frustration and disillusionment, potentially causing burnout. This relationship appears bidirectional, with curiosity influencing burnout and burnout and stress also affecting the ability to connect with curiosity.

### Category III: curiosity’s significance in medical education from the learner’s and teacher’s perspective

#### Theme: physicians lament a lack of curiosity in students

Many doctors noted a lack of motivation among medical students, with some attributing it to compulsory courses and the high workload in medical studies. Furthermore, they observed that different student aptitudes result in varying levels of curiosity about the subjects taught, leading to a lack of interest and distraction during lectures. Additionally, one participant assessed a generational shift, with students and younger doctors prioritizing work-life balance over working overtime.

#### Theme: curiosity in medical students and physicians can be nurtured with the right methods and in the right conditions

Most physicians regarded it possible to cultivate curiosity in students. They suggested that clinical activities, particularly direct patient contact, would arouse curiosity. Interactive teaching formats and easy access to information, such as *via* the internet, were emphasized. The school-like nature of medical studies and the lack of choice for students were seen as negative influences. Physicians expressed that medical students should have more opportunities to set their own priorities and pursue their own interests in a curiosity-driven manner. For nurturing curiosity in the medical profession, collegial exchange, both in formal settings, such as in further training, conferences, or internal departmental meetings, and informal discussions among colleagues, were viewed as valuable. The availability of time emerged as crucial for facilitating or impeding such exchanges, often challenged by the demanding daily workload. Rigid hierarchies, common in clinical medicine, were identified as another negative factor that hinders collegial exchange.

## Discussion

### Main findings

The study explored physicians’ perspectives on the significance of curiosity in their professional practice and medical training. Interviewees highlighted three crucial aspects of curiosity: its role in lifelong education, its impact on building empathetic physician-patient relationships, and its importance as a quality for effective research. Physicians linked curiosity to positive emotions and its inhibition to negative emotions, often attributing external factors, such as time constraints and lack of autonomy, to hindering their curiosity. Despite these challenges, physicians believed in fostering curiosity through methods like interactive learning and exposure to new challenges. Opinions varied regarding the relationship between burnout and curiosity: While some physicians argued that curiosity can serve as a protective factor against burnout, others viewed excessive curiosity as a potential source of frustration and burnout.

### Curiosity’s role in physicians’ work

The interviewees attributed a wide range of positive effects to curiosity. They viewed curiosity as a driver for knowledge acquisition aligning with research in other areas that highlight its positive impacts in the academic setting [6–8] such as in knowledge acquisition [9–11] and lifelong learning [32]. Furthermore, the importance of curiosity in physician-patient relationships and as a quality of a good researcher was emphasized, validating similar sentiments shared by medical teachers and researchers [28,32,33]. It is noteworthy that many physicians highlighted its importance in handling unusual clinical cases, which require a combination of broad medical knowledge and a curious mindset. This has not been previously discussed in the literature.

### What influences curiosity?

The results of this study raise an intriguing question about the evolution of curiosity in physicians over time. Participants generally agreed that curiosity undergoes transformation, tending to become more specialized within their respective fields, but had different opinions on whether curiosity in total increased or decreased in the workplace. While there is a scarcity of empirical studies measuring physicians’ curiosity over time, a study on medical students found a significantly lower intellectual medical curiosity in final year students compared to first year students [42].

Physicians had strong intuitions about the factors that influenced their curiosity: diverse activities, having sufficient time, professional exchanges with colleagues, and facing new challenges were seen as fostering curiosity, while rigid hierarchies and a lack of autonomy were identified as stifling curiosity and impeding its pursuit. The detrimental effects of stress and time constraints on curiosity were notably emphasized, suggesting an area that needs addressing, especially given the high levels of stress and workload reported by German physicians [43,44]. Current models of curiosity promotion emphasizing variety and positive challenges [45,46] are supported by the findings of this study. In addition, they suggest practical approaches to fostering curiosity in physicians and indicate potential avenues for future research, especially since there don’t exist any quantitative studies on how to promote curiosity in physicians or other cohorts.

In medical education, participants felt that hands-on, interactive teaching methods positively influenced their own curiosity and that of students. Research shows that active learning formats, such as problem-based-learning and flipped classrooms, are well received by students [47] and that students learn more than in lecture-based teaching [48,49]. The results of this study suggest that these may also positively impact student motivation and curiosity, warranting further investigation. While many existing curricula [50] already reflect active learning and practice-based teaching methods, such as skills labs, there is potential to improve and expand these approaches.

### Curiosity and well-being

Participants in the study overwhelmingly associated curiosity with positive emotions such as enjoyment and satisfaction. In contrast, inhibiting curiosity or working in an environment that didn’t stimulate it was linked to dissatisfaction, boredom, and exhaustion. One person even went so far as to associate it with depression. This is consistent with studies in other populations, where high curiosity correlates with a range of positive mental health outcomes: greater life satisfaction and meaning in life [21,22,51], positive relationship outcome [52], proactive socialization behaviors [17], and well-being and health [18]. An intriguing finding of this study was a reciprocal relationship between curiosity and stress: some physicians viewed the suppression of curiosity as a source of stress, while elevated stress levels were perceived to reduce curiosity.

Burnout among physicians is a pervasive issue, with rates that are both high and continuing to rise [15,16]. Therefore, the participants’ differing views on the relationship between curiosity and burnout were particularly interesting. Some physicians saw curiosity as a protective factor, shielding against burnout through intrinsic motivation, while others saw excessive curiosity (which ultimately cannot be satisfied) as a possible cause of frustration and thus inducing burnout. Distinguishing between a more ‘dysfunctional’ and a more ‘functional’ curiosity might explain why some types of curiosity may exacerbate stress and burnout, while others may protect against them. It would be valuable to gain a clearer understanding of how burnout affects curiosity, as one qualitative study reported that physicians felt burnout significantly diminished their curiosity to learn [53]. Despite much anecdotal evidence, no study has directly investigated the relationship between curiosity and burnout. However, a 2015 study [54] shows that personal initiative fully mediates the relationship between curiosity and emotional exhaustion, a key indicator of employees’ quality of work life and an aspect of the burnout syndrome. Further studies are needed to clarify this relationship.

### Limitations and strengths

Despite its innovative character, this study has several limitations. All participating physicians worked at the same University Hospital. Consequently, the opinions and experiences of doctors in a large hospital, in a high-income country, with research and teaching responsibilities are the only ones reflected in the results. However, this also allows for clear comparisons of the physicians’ statements and strengthens the conclusions drawn from this study in this particular cohort. This selection also ensured that the qualitative data in this study was actually based on the experience of clinicians working in a hospital, which should be considered a strength of the study. Additionally, the researchers intentionally avoided presenting respondents with an overly specific definition of curiosity. This approach, while a strength in revealing interesting and varied insights, means that statements might have been based on slightly different mental models. Furthermore, it is important to consider that the interviewees might have been influenced by the framing of the questions, some of which were dichotomous, or responded in a socially desirable manner, providing answers they believe the researchers want to hear. Finally, every study must strike a balance between the breadth and the depth of the topics investigated. Since this is one of the first studies on physician curiosity, the authors intentionally chose to cover a broad range of topics rather than delving deeply into one area. This approach limits the amount of information that can be gathered, and future research should focus on exploring specific aspects of curiosity in greater detail.

### Summary and directions for future research

This study found that physicians overwhelmingly regard curiosity as a positive trait, essential for both personal and professional development and improving patient care. The physicians’ views align with research in other areas that links the fulfillment of curiosity with positive emotions and its frustration with negative emotions. Key factors influencing curiosity were identified, with time pressure and stress being notable hindrances. These insights are relevant for hospital administrators and other stakeholders interested in promoting curiosity within the medical profession. The study lays the groundwork for several potential research directions, particularly considering the participants’ diverse opinions on the evolution of curiosity over time and its potential relationship with burnout. Further exploration of these topics is necessary. Moreover, this study highlights the necessity for more extensive investigations, both qualitative and quantitative, into the impact of curiosity in clinical medicine and research. Future research would benefit from larger studies using mixed methods in various hospitals and regions. Exploring the factors that promote or inhibit curiosity among physicians and medical students can enhance the insights provided by this study, offering a more nuanced understanding of the role of curiosity in the medical field.

## Conclusion

To borrow a metaphor from one interviewee: Is curiosity a flame that, if kindled and nurtured, can ignite a passion in physicians and serve as a driving force? How is it kindled, and could it also cause harm in the wrong circumstances or if used incorrectly? What does it mean for doctors’ mental health when this flame burns brightly or is suffocated? Could the extinguishing of curiosity’s fire even be a sign of burnout syndrome? The study provides insights into these considerations and encourages readers to think about how to better nurture curiosity in physicians. The findings suggest that while curiosity may play an important role in medical professionalism and patient care, it is often hindered by working conditions and could be fostered better. Additionally, this study enhances the understanding of physician curiosity and raises important new research questions about the value of curiosity to medical professionalism, patient care, and physician well-being.

## Supplementary Material

COREQ checklist_3rd revision.pdf

## Data Availability

The data that support the findings of this study are available from the corresponding author, TJB, upon reasonable request.
